# Association of parental verbal responsivity with reduced mental health problems in preschool-aged children: a cross-sectional study of 21,366 dyads in western China

**DOI:** 10.3389/fpsyg.2026.1688294

**Published:** 2026-02-05

**Authors:** Fenling Feng, Hongli Sun, Yujun Wang

**Affiliations:** 1 Xi'an International Medical Center Hospital, Xi'an, Shaanxi, China; 2 Tianyou Children's Hospital of Chang'an, Xi'an, Shaanxi, China; 3 Shaanxi Institute for Pediatric Diseases, Xi'an Key Laboratory of Children's Health and Diseases, Xi'an Children's Hospital, National Regional Children's Medical Center (Northwest), Xi'an, Shaanxi, China; 4 Department of Traditional Chinese Medicine and Western Medicine, Xi'an Children's Hospital, Affiliated Children's Hospital of Xi'an Jiaotong University, National Regional Children's Medical Center (Northwest), Xi'an, Shaanxi, China

**Keywords:** cross-sectional study, mental health problems, parental verbal responsivity, positive communication, preschool-aged children

## Abstract

**Background:**

Mental health problems affect 7%−18% of preschool-aged children globally, with early interventions crucial for mitigating long-term impacts. Parental verbal responsivity (PVR) is a modifiable factor linked to cognitive and emotional development, yet large-scale studies on its association with mental health in young children remain scarce.

**Methods:**

This cross-sectional study was conducted between February 28 and March 5, 2025, in a western Chinese city, involving 21,366 parent-child dyads from 189 kindergartens. Parental Verbal Responsivity (PVR) was assessed using the parent-reported StimQ, which measures verbal interactions during daily routines, play, and regulation activities. Child mental health was evaluated via the parent-reported Strengths and Difficulties Questionnaire (SDQ). Multivariable logistic regression models were employed, adjusting for relevant sociodemographic and lifestyle covariates.

**Results:**

After full adjustment, each unit increase in PVR corresponded to 4% reduced odds of total difficulties (95%CI 0.95–0.96) and 7% increased odds of prosocial behaviors (95%CI 1.06–1.08), both with *P* < 0.0001 significance. Subgroup analyses revealed effect modifications by parental gender, education, employment status, annual family income, and smoking status and alcohol intake status.

**Conclusion:**

In this large-scale study, a weak association was found between verbal interaction frequency and child mental health, explaining less than 1% of outcome variance. This highlights the limitation of measuring only interaction frequency and underscores the need for future research to incorporate multi-informant and observational methods to better understand pathways to child mental health.

## Background

Although mental health problems can arise throughout childhood, the preschool years (ages 3–6) constitute a particularly sensitive and unique developmental window, during which emotional and behavioral difficulties—most commonly hyperactivity/inattention, emotional symptoms, conduct problems, and peer relationship problems—frequently first become apparent. Reported prevalence rates of behavioral and emotional problems in preschool children vary across Asian populations, ranging from 11.9% to 25.1%, with higher rates often linked to specific demographic or socioeconomic factors (Teekavanich et al., 2017; Wu et al., 2012). However, large-scale, population-representative epidemiological data on preschool mental health remain scarce in western China. Longitudinal research indicates that symptoms of poor mental health can emerge in infancy and persist across childhood and adolescence into adulthood, influenced by individual and familial trajectories (Nilsen et al., 2017; Steinvoord and Junge, 2019). These conditions are associated with adverse outcomes, including diminished academic performance (Gräf et al., 2019) and increased risk of communicable and non-communicable diseases (Prince et al., 2007). Early identification and intervention are critical for mitigating these impacts, improving developmental outcomes, and enhancing quality of life (McGorry and Mei, 2018). Among the multiple influences on preschool children's mental health, the family caregiving environment represents one of the most proximal and modifiable environmental factors. Within this caregiving context, parental verbal responsivity (PVR)—the frequency, contingency, and emotional warmth of parents' verbal responses to their child during daily routines, play, and emotion-regulation moments—stands out as a key protective mechanism. Therefore, investigating PVR is of paramount importance for developing evidence-based preventive strategies. PVR refers to a parent's responsive, non-structured verbal interactions with their child, such as engaging in games like peek-a-boo, bath play, or talking about the day's events (Dreyer et al., 1996). It measures how parents spontaneously use language to connect with and stimulate their child's cognitive development. Previous research has consistently shown that high-quality parent–child interactions during everyday activities can significantly enhance parent–child relationship closeness (intimacy) and attachment security. Examples of such interactions include joint play (Teufl and Ahnert, 2022), parent-implemented early language and communication interventions (Cocquyt et al., 2024), and shared home learning activities (e.g., painting, playing games, reading together, singing, and teaching letters and numbers) (Hoyne and Egan, 2024). High-quality parent–child interactions foster closeness and secure attachment through contingent, warm verbal exchanges. This association is supported by attachment theory (secure base formation), Vygotsky's sociocultural theory (scaffolding in the zone of proximal development), Bronfenbrenner's bioecological model (proximal processes as primary developmental drivers), and the transactional model (bidirectional cycles of responsiveness). Collectively, these frameworks indicate that higher PVR promotes emotional security, self-regulation, and prosocial behavior while reducing mental health risks in preschool children.

Seminal work by Hart and Risley underscores the critical role of verbal exposure in early vocabulary development, highlighting that the quantity of words a child hears and the degree of parental encouragement before age three are pivotal environmental predictors (Freeman and Kasari, 2013). However, parental communication styles vary: responsive strategies, such as linguistic mapping and expansions, foster child-initiated communication, whereas directive approaches, which redirect attention or demand behavioral changes, may hinder linguistic growth (Edmunds et al., 2019). PVR is a key facilitator of early language development, a period marked by rapid linguistic advancement. The mechanisms underlying PVR's influence on language outcomes may hinge on whether parents respond directly to children's communicative acts or merely to their focus of attention (Shriver et al., 2020). Responsive PVR fosters a transactional cycle, in which improved language abilities facilitate more intricate forms of interaction, which additionally supports the growth of language proficiency (Hunter and Daw, 2021). To assess PVR, this study employs the validated StimQ assessment, which quantifies verbal interactions across three behavioral domains: daily routines, imaginative play, and regulatory activities (Dreyer et al., 1996; Mendelsohn et al., 1999). The PVR evaluates cognitive stimulation in the home environment, focusing on verbal exchanges during routine activities, such as conversational exchanges during feeding or joint sound-making (Dreyer et al., 1996; Mendelsohn et al., 1999).

Although a limited number of studies have directly examined the association between PVR and preschool children's mental health, accumulating evidence indicates that higher levels of responsive, contingent verbal interaction are consistently linked to better emotional and behavioral outcomes. Specifically, greater PVR—characterized by frequent conversational turns, affective verbal engagement, linguistic mapping, and verbal scaffolding during daily routines, play, and emotion-regulation activities—has been associated with higher self-esteem, fewer internalizing and externalizing problems, reduced anxiety symptoms, and enhanced prosocial behavior in young children (Lee and Ha, 2023; Polcari et al., 2014; Solomon and Serres, 1999). In contrast, low parental verbal responsivity, manifested as infrequent or non-contingent verbal exchanges without hostility, is related to poorer emotion regulation, increased behavioral difficulties, and lower prosocial scores, independent of overt verbal aggression (Percy et al., 2016). Notably, a significant gap exists in large-scale research on PVR and mental health outcomes specifically for children aged 3–6 years. To address this, our study leverages a large, population-based cohort from a Chinese city, employing stratified cluster sampling across 189 kindergartens to ensure diverse representation and generate robust evidence on PVR's impact in this critical developmental window. To accurately isolate this impact from confounding contextual factors, our analysis incorporates key variables—socioeconomic status (SES), parental substance use, and educational attainment—whose influence is well-established. Extant literature consistently links low SES and financial strain to increased family stress and compromised caregiving (Nilsen et al., 2025), while parental cigarette and alcohol use is directly associated with reduced maternal sensitivity and child emotional dysregulation (Kisner et al., 2025; Wang et al., 2025). Educational attainment further intersects with these factors, affecting parenting resources and knowledge (Sajewicz-Radtke et al., 2025; Liu et al., 2025). Including these variables is therefore essential to disentangle the specific mechanistic pathway from contextual adversity to PVR and, ultimately, to child mental health.

## Materials and methods

### Study population

This study employed a cross-sectional study design, jointly implemented by a municipal education administration and local educational institutions in a western Chinese city. Using multistage probability proportional to size sampling, we selected 13 administrative divisions as primary sampling units, with study sites systematically and randomly chosen from the official kindergarten registry in each district. The research adopted a stratified cluster random sampling strategy, applying population-proportionate sampling to ensure the sample size was proportional to the population of enrolled preschool children aged 3–6 years in each district, thereby enhancing generalizability. The final cohort included 189 preschool education institutions, representing various levels of urban-rural dichotomy. Study participants comprised children in junior (3–4 years), middle (4–5 years), and senior (5–6 years) classes and their primary caregivers. Data collection was conducted through standardized questionnaire surveys between February 28 and March 5, 2025. Following the informed consent principle, the institution-assisted survey was administered after obtaining written informed consent forms from guardians. Using nuclear family sampling methodology, one legal guardian per household served as a proxy respondent to complete the assessment. The initial study cohort included 25,017 parent-child dyads. After data cleaning procedures and application of predetermined exclusion criteria, we excluded: (1) responses from non-parental guardians (*n* = 2,408) and (2) cases with missing critical demographic variables, including missing caregiver age (*n* = 517) and missing child age (*n* = 726). The final analytic dataset contained 21,366 valid cases, achieving a valid response rate of 85.41%. The study protocol strictly adhered to the ethical principles of the Declaration of Helsinki and received ethical approval from the Institutional Review Board of Xi'an Jiaotong University Children's Hospital (Approval No. 20250225-21).

### Assessments of the children's mental health problems

Parents provided standardized assessments of their children's psychological wellbeing through the official simplified Chinese version of the Strengths and Difficulties Questionnaire (SDQ) and the SDQ's psychometric properties remain robust across its 40+ language versions and wide age applicability (3–17 years) (Goodman and Goodman, 2009; Du et al., 2008; Pang et al., 2024). This 25-item instrument, employing a 3-point Likert scale (0 = Not True to 2 = Certainly True), represents the gold standard for developmental behavioral screening in epidemiological studies. The items can be divided into the following subscales: emotional symptoms, conduct problems, peer relationship problems, hyperactivity/inattention, and prosocial behavior. The SDQ prosocial scale is a stand-alone measure of positive emotions and behaviors toward others, representing the “strengths” portion of the SDQ (Zoumenou et al., 2025). Each subscale yields scores from 0 to 10, while the first four symptom subscales combine for a total difficulties score (0–40); using Chinese-recommended cutoffs, we dichotomized outcomes as normal ( ≤ 14) vs. at-risk (>14) and identified potential prosocial deficits at scores < 6 (Wang et al., 2024). This internationally recognized screening measure allows parents to systematically document clinically relevant aspects of their child's socioemotional development (Goodman, 1997).

### Assessments of parental verbal responsivity

Parental verbal responsivity (PVR) was assessed using the StimQ, which is a validated ecological assessment tool specifically designed for children aged 0–6 years (Salve et al., 2023). The StimQ measures four distinct domains: READ (shared book reading), PIDA (parental involvement in developmental activities), PVR (parental verbal interactions), and ALM (availability of learning materials). In this study, the PVR scale was administered as a parent-report instrument, assessing three aspects of parental verbal interactions: everyday routines (0–9 points), play, pretend, and imagination (0–6 points), and regulation activities (0–4 points). These scores were combined into a composite PVR score (0–19 points), with higher values indicating stronger parental verbal responsivity (Choi et al., 2018).

### Variables

The variable in this study include child age, child gender, age, gender, education level, employment status, marital status, annual family income, smoking status, and alcohol intake status. Participants were categorized by age into distinct groups: children (< 4 years, 4–5 years, and ≥5 years) and parents (< 30 years, 30–45 years, and ≥45 years), with gender recorded as male or female for both groups. Parental education attainment was stratified into three tiers: elementary education or below ( ≤ primary school), secondary education (junior/senior high school), and post-secondary qualifications (≥junior college). Employment status was dichotomized into working and not working. Marital status was categorized as married or other, and annual family income was grouped into < ¥60,000, ¥60,000 to ¥150,000, and >¥150,000. Smoking status was classified into three groups: never smokers, former smokers, and current smokers. Similarly, alcohol intake status was grouped as never drinkers, former drinkers, and current drinkers.

### Statistical analysis

All analyses were performed using EmpowerStats (https://www.empowerstats.net/cn/) and the statistical package R (4.2 version). Continuous variables expressed as mean ± standard deviation and categorical variables presented as percentages (%). Chi-square tests were used to compare categorical variables, with statistical significance set at *P* < 0.05. Multivariable logistic regression models were employed to evaluate the association between PVR and children's mental health problems, including both unadjusted models and adjusted models controlling for potential confounders (child age and gender, parental age and gender, education level, employment status, marital status, annual family income, smoking status, and alcohol intake status). Additionally, subgroup analyses and interaction tests were conducted to assess potential effect modification by these covariates on the PVR-mental health relationship.

## Results

### Baseline characteristics

This cross-sectional study initially enrolled 25,017 parent-child pairs. After applying exclusion criteria, the final cohort comprised 21,366 participants, including 11,062 males and 10,304 females. Participant characteristics showed mean ages of 34.75 ± 4.56 years for parents and 4.81 ± 0.89 years for their children. Participant characteristics across different levels of total difficulties are detailed in Table 1. Significant intergroup differences emerged across all measured variables (all *P* < 0.05), with the exception of child age which showed no statistical significance (*P* > 0.05). Compared with the lower group (total difficulties score ≤ 14), participants in the higher group (total difficulties score >14) were generally younger and had lower PVR A subscale, PVR B subscale, PVR C subscale and PVR scale.

**Table 1:** Baseline characteristics of the study participants according to total difficulties score.

**Mean** + **SD/N (%)**		**Total difficulties score ≤ 14 (*n* = 17,388)**	**Total difficulties score >14 (*n* = 3,978)**	***P*-value**
Child age (years)	4.82 ± 0.89	4.82 ± 0.88	4.08 ± 0.90	0.256
Child gender				< 0.001
Boys	11,062 (51.77%)	8,897 (51.17%)	2,165 (54.42%)	
Girls	10,304 (48.23%)	8,491 (48.83%)	1,813 (45.58%)	
Parent age (years)	34.75 ± 4.56	34.81 ± 4.53	34.48± 4.68	< 0.001
Parent gender				< 0.001
Men	5,108 (23.91%)	3,961 (22.78%)	1,147 (28.83%)	
Female	16,258 (76.09%)	13,427 (77.22%)	2,831 (71.17%)	
Education level				< 0.001
≤ Primary school	1,479 (6.92%)	1,039 (5.98%)	440 (11.06%)	
Junior high school and senior high school	8,355 (39.10%)	6,651 (38.25%)	1,704 (42.84%)	
≥Junior College	11,532 (53.97%)	9,698 (55.77%)	1,834 (46.10%)	
Employment status				< 0.001
Working	15,642 (73.21%)	12,912 (74.26%)	2,730 (68.63%)	
Not working	5,724 (26.79%)	4,476 (25.74%)	1,248 (31.37%)	
Marital status				< 0.001
Married	20,720 (96.98%)	16,906 (97.23%)	3,814 (95.88%)	
other	646 (3.02%)	482 (2.77%)	164 (4.12%)	
Annual family income, in thousands, ¥				< 0.001
< 60	13,024 (60.96%)	10,261 (59.01%)	2,763 (69.46%)	
≤ 60 to 150	6,791 (31.78%)	5,775 (33.21%)	1,016 (25.54%)	
≥150	1,551 (7.26%)	1,352 (7.78%)	199 (5.00%)	
Smoking status				< 0.001
Never	18,187 (85.12%)	14,952 (85.99%)	3,235 (81.32%)	
Ex-smoker	478 (2.24%)	374 (2.15%)	104 (2.61%)	
Current	2,701 (12.64%)	2,062 (11.86%)	639 (16.06%)	
Alcohol intake status				< 0.001
Never	17,858 (83.58%)	14,718 (84.64%)	3,140 (78.93%)	
Ex - drinker	1,003 (4.69%)	748 (4.30%)	255 (6.41%)	
Current	2,505 (11.72%)	1,922 (11.05%)	583 (14.66%)	
PVR scale score	14.11 ± 4.25	14.30 ± 4.11	13.28 ± 4.75	< 0.001

Mean ± SD for continuous variables: *P* value was calculated by *t* test. % for categorical variables: *P* value was calculated by chi-square test.

### Relationship between parental verbal responsivity and mental health problems

Based on the results presented in Table 2, PVR demonstrated a consistent, statistically significant association with both measures of child mental health, both before and after adjusting for relevant sociodemographic and lifestyle covariates. For total difficulties, a one-unit increase in PVR score was associated with a 4% reduction in the odds of being categorized as at-risk (adjusted OR = 0.96, 95% CI: 0.95–0.96, *p* < 0.0001). When analyzed as a continuous outcome, the same increase in PVR corresponded to a significant decrease in the total difficulties score (adjusted β = −0.08, 95% CI: −0.10 to −0.07, *p* < 0.0001). For prosocial behavior, the association was positive and slightly stronger. A one-unit increase in PVR score was linked to a 7% increase in the odds of having a higher level of prosocial behavior (adjusted OR = 1.07, 95% CI: 1.06–1.08, *p* < 0.0001). Similarly, when treated continuously, higher PVR was associated with a significant increase in the prosocial behavior score (adjusted β = 0.09, 95% CI: 0.08–0.09, *p* < 0.0001). Stratified analyses and interaction tests (Tables 3, 4) revealed effect modifications in the association between PVR and mental health outcomes. For total difficulties, the composite PVR score showed significant interactions with smoking status (P interaction = 0.0286) and alcohol intake status (P interaction = 0.0149).

**Table 2:** Relationship between parental verbal responsivity and mental health problems.

**Exposure**	**Non-adjusted**	**Adjust**
**Total difficulties (categorical)**		
PVR scale score	0.95 (0.94, 0.96) < 0.0001	0.96 (0.95, 0.96) < 0.0001
**Total difficulties score (continuous)**		
PVR scale score	−0.10 (−0.12, −0.09) < 0.0001	−0.08 (−0.10, −0.07) < 0.0001
**Prosocial behavior (categorical)**		
PVR scale score	1.07 (1.06, 1.08) < 0.0001	1.07 (1.06, 1.08) < 0.0001
**Prosocial behavior score (continuous)**		
PVR scale score	0.09 (0.08, 0.09) < 0.0001	0.09 (0.08, 0.09) < 0.0001

Results: β (95%CI) *P* value/OR (95%CI) *P* value. Non-adjusted model adjust for: None. Adjust model adjust for: Child age, child gender, parent age, parent gender, education level, employment status, marital status, annual family income, smoking status, and alcohol intake status.

**Table 3:** Stratified analysis between parental verbal responsivity and total difficulties.

**Total difficulties**	**PVR scale**	**P interaction**
Child age (years)		0.0974
< 4	0.95 (0.93, 0.96) < 0.0001	
≥4, < 5	0.95 (0.94, 0.96) < 0.0001	
≥5	0.96 (0.95, 0.98) < 0.0001	
Child gender		0.6539
Boys	0.95 (0.94, 0.96) < 0.0001	
Girls	0.96 (0.95, 0.97) < 0.0001	
Parent age (years)		0.5126
< 30	0.95 (0.93, 0.97) < 0.0001	
≥30, < 45	0.96 (0.95, 0.97) < 0.0001	
≥45	0.97 (0.92, 1.02) 0.1896	
Parent gender		0.0500
Men	0.97 (0.95, 0.98) < 0.0001	
Female	0.95 (0.94, 0.96) < 0.0001	
Education level		0.4545
≤ Primary school	0.96 (0.94, 0.98) 0.0006	
Junior high school and senior high school	0.96 (0.95, 0.97) < 0.0001	
≥Junior college	0.95 (0.94, 0.96) < 0.0001	
Employment status		0.1464
Working		
Not working	0.95 (0.94, 0.96) < 0.0001	
Marital status		0.4094
Married	0.96 (0.95, 0.96) < 0.0001	
other	0.94 (0.91, 0.98) 0.0014	
Annual family income, in thousands, ¥		0.3168
< 60	0.96 (0.95, 0.97) < 0.0001	
≤ 60 to 150	0.95 (0.93, 0.96) < 0.0001	
≥150	0.98 (0.94, 1.02) 0.2868	
Smoking status		0.0286
Never	0.95 (0.94, 0.96) < 0.0001	
Ex-smoker	0.94 (0.89, 0.99) 0.0182	
Current	0.98 (0.96, 1.00) 0.0669	
Alcohol intake status		0.0149
Never	0.95 (0.94, 0.96) < 0.0001	
Ex-drinker	0.96 (0.93, 0.99) 0.0138	
Current	0.98 (0.96, 1.01) 0.1516	

Restults: OR (95%CI) *P* value. Adjusted for: Parent age, parent gender, education level, employment status, annual family income, smoking status, alcohol intake status, and marital status, along with child age and gender, except for the variable used in each stratified analysis.

**Table 4:** Stratified analysis between parental verbal responsivity and prosocial behavior.

**Prosocial behavior**	**PVR scale**	***P* interaction**
Child age (years)		0.8081
< 4	1.07 (1.05, 1.08) < 0.0001	
≥4, < 5	1.07 (1.06, 1.08) < 0.0001	
≥5	1.07 (1.06, 1.08) < 0.0001	
Child gender		0.9251
Boys	1.07 (1.06, 1.08) < 0.0001	
Girls	1.07 (1.06, 1.08) < 0.0001	
Parent age (years)		0.3422
< 30	1.08 (1.06, 1.10) < 0.0001	
≥30, < 45	1.07 (1.06, 1.08) < 0.0001	
≥45	1.05 (1.01, 1.09) 0.0203	
Parent gender		0.3576
Men	1.07 (1.06, 1.09) < 0.0001	
Female	1.07 (1.06, 1.07) < 0.0001	
Education level		0.0991
≤ Primary school	1.05 (1.03, 1.08) < 0.0001	
Junior high school and senior high school	1.06 (1.05, 1.07) < 0.0001	
≥Junior college	1.08 (1.07, 1.09) < 0.0001	
Employment status		0.9623
Working	1.07 (1.06, 1.08) < 0.0001	
Not working	1.07 (1.06, 1.08) < 0.0001	
Marital status		0.7595
Married	1.07 (1.06, 1.08) < 0.0001	
other	1.06 (1.03, 1.10) 0.0005	
Annual family income, in thousands, ¥		0.4114
< 60	1.07 (1.06, 1.07) < 0.0001	
≤ 60 to 150	1.08 (1.06, 1.09) < 0.0001	
≥150	1.07 (1.04, 1.10) < 0.0001	
Smoking status		0.7201
Never	1.07 (1.06, 1.08) < 0.0001	
Ex-smoker	1.08 (1.03, 1.14) 0.0024	
Current	1.06 (1.04, 1.08) < 0.0001	
Alcohol intake status		0.1771
Never	1.07 (1.06, 1.08) < 0.0001	
Ex-drinker	1.06 (1.03, 1.09) 0.0003	
Current	1.05 (1.03, 1.07) < 0.0001	

Restults: OR (95%CI) *P* value. Adjusted for: parental gender, age, education level, employment status, annual family income, smoking status, alcohol intake status, and marital status, along with child age and gender, except for the variable used in each stratified analysis.

## Discussion

This large cross-sectional study of 21,366 parent–child dyads in western China found statistically significant but very small associations between parent-reported parental verbal responsivity (PVR) and parent-reported mental health outcomes in preschool children. After full adjustment, each 1-point increase in the PVR composite score was associated with a 4% lower odds of total difficulties (adjusted OR 0.96) and a 7% higher odds of prosocial behavior (adjusted OR 1.07). Although these associations are highly significant (*P* < 0.0001), the effect sizes are extremely small (ORs very close to 1.00). At the individual child level, therefore, the clinical relevance of differences in PVR score is likely to be modest, and the observed associations should not be overstated. Crucially, the most plausible explanation for the observed marginal correlation is Common Method Variance (CMV). Both the predictor (StimQ-PVR) and the outcome (SDQ) were derived from the same informant (the parent). A parent's current psychological state, overall reporting style, or general perception of the parent-child relationship could easily account for the shared variance of less than 1% between their assessment of their own verbal behavior and their rating of their child's behavior. Therefore, the detected association likely reflects shared rater bias rather than a direct causal mechanism linking verbal frequency to mental health. This fundamental limitation tempers any causal interpretation of the results.

The study's use of the StimQ-PVR subscale, which evaluates verbal interactions across everyday routines, imaginative play, and regulatory activities, provides a nuanced understanding of how specific domains of PVR contribute to mental health in toddlers and children (Malhi et al., 2018; Jimenez et al., 2021; Green et al., 2009). PVR is a core factor for children's language and socio-emotional development. PVR varies by context and adapts to a child's developmental level. For example, book sharing and social play elicit higher rates of child communication and more supportive parental responses (Lee and Ha, 2023; Laudańska et al., 2025; Dyne et al., 2025). In clinical populations, such as children with autism or developmental delay, parents often use fewer language-enhancing responses like expansions and follow-in directives, which are strongly predictive of later language outcomes (Grzadzinski et al., 2021; Delehanty et al., 2024a,b). Maternal mental health also influences PVR: mothers with postpartum psychosis show significantly reduced verbal output and responsiveness (Raju et al., 2025). The primary value of this study lies not in identifying a powerful intervention target but in providing a robust, large-scale descriptive benchmark. It quantifies the tenuous nature of the link between a simple measure of verbal interaction frequency and complex mental health outcomes as reported by the same parent. This finding ‘calibrates expectations' in the field, demonstrating that such frequency measures, while convenient for large-scale surveys, have limited standalone explanatory power. Future research must move beyond simplistic frequency counts. To understand if and how verbal interactions matter, studies should prioritize: (1) multi-informant designs (e.g., incorporating teacher reports or observational data) to overcome CMV; (2) direct assessment of interaction quality, affective tone, and contingency; and (3) investigation of moderators (e.g., socioeconomic status, child temperament) that might reveal contexts where interaction patterns are more impactful.

Subgroup analyses indicated that the small associations were modestly modified by parental smoking and alcohol-use status, suggesting that lifestyle-related family stress or reduced parental availability may attenuate any potential benefit of verbal interaction frequency. These interaction effects further highlight the embeddedness of PVR within broader socioeconomic and familial contexts.

### Limitations

Although this study provides valuable insights, several limitations should be noted. These limitations also highlight clear directions for future research. First, the cross-sectional design prevents causal inferences. Longitudinal studies are needed to clarify the causal and dynamic relationships between parental verbal responsivity (PVR) and children's psychological and behavioral outcomes. Second, both PVR (measured by the StimQ) and child behavioral outcomes (measured by the SDQ) relied exclusively on parent-reported instruments. This approach, while practical for large-scale studies, may introduce measurement bias due to variations in parental perceptiveness, education level, and reporting tendencies. Furthermore, using the same informant for both exposure and outcome increases the risk of common-method variance, potentially inflating observed associations. Future research would benefit from incorporating standardized behavioral observations, multi-informant reports (e.g., from teachers or caregivers), or objective measures to enhance the robustness and validity of findings. Third, although the sample was large and drawn from a major metropolitan area in China, generalizability to rural populations, less-developed regions, or different cultural contexts should be interpreted with caution. In summary, the present findings should be regarded as preliminary evidence, and addressing these limitations in future work will strengthen our understanding of how parental verbal responsivity influences child development.

## Conclusion

In summary, this large cross-sectional study documents a statistically robust yet substantively very weak association between parent-reported frequency of verbal interactions and parent-reported mental health in preschool children. Given the minimal effect sizes, the exclusive reliance on single-informant reports, and the absence of data on interaction quality, these findings do not support isolated frequency of verbal exchange as a meaningful clinical or public health intervention target. Instead, the study provides a definitive quantitative reference point for the strength of this relationship in a large population, underscoring the critical need for more nuanced, multi-method longitudinal research to identify the specific components of parent-child interaction that may yield clinically meaningful benefits for mental health.

## Data Availability

The original contributions presented in the study are included in the article/supplementary material, further inquiries can be directed to the corresponding author.
